# Semaglutide in HFpEF across obesity class and by body weight reduction: a prespecified analysis of the STEP-HFpEF trial

**DOI:** 10.1038/s41591-023-02526-x

**Published:** 2023-08-27

**Authors:** Barry A. Borlaug, Dalane W. Kitzman, Melanie J. Davies, Søren Rasmussen, Eric Barros, Javed Butler, Mette Nygaard Einfeldt, G. Kees Hovingh, Daniél Vega Møller, Mark C. Petrie, Sanjiv J. Shah, Subodh Verma, Walter Abhayaratna, Fozia Z. Ahmed, Vijay Chopra, Justin Ezekowitz, Michael Fu, Hiroshi Ito, Małgorzata Lelonek, Vojtech Melenovsky, Julio Núñez, Eduardo Perna, Morten Schou, Michele Senni, Peter van der Meer, Dirk Von Lewinski, Dennis Wolf, Mikhail N. Kosiborod

**Affiliations:** 1 Department of Cardiovascular Medicine, Mayo Clinic, Rochester, MN USA; 2 Department of Cardiovascular Medicine and Section on Geriatrics and Gerontology, Wake Forest University School of Medicine, Winston-Salem, NC USA; 3 Diabetes Research Centre, University of Leicester, Leicester, UK; 4 NIHR Leicester Biomedical Research Centre, Leicester, UK; 5 Novo Nordisk A/S, Søborg, Denmark; 6 Baylor Scott and White Research Institute, Dallas, TX and Department of Medicine, University of Mississippi, Jackson, MS USA; 7 School of Cardiovascular and Metabolic Health, University of Glasgow, Glasgow, UK; 8 Division of Cardiology, Department of Medicine, Northwestern University Feinberg School of Medicine, Chicago, IL USA; 9 Division of Cardiac Surgery, Li Ka Shing Knowledge Institute of St. Michael’s Hospital, Unity Health Toronto, University of Toronto, Toronto, ON Canada; 10 College of Health and Medicine, The Australian National University, Canberra, ACT Australia; 11 Division of Cardiovascular Sciences, Faculty of Biology, Medicine and Health, University of Manchester, Manchester, UK; 12 Clinical Cardiology, Heart Failure and Research, Max Super Specialty Hospital, Saket, New Delhi, India; 13 University of Alberta, Edmonton, AB Canada; 14 Section of Cardiology, Department of Medicine, Sahlgrenska University Hospital-Ostra, Gothenburg, Sweden; 15 Department of General Internal Medicine 3, Kawasaki Medical School, Okayama, Japan; 16 Department of Noninvasive Cardiology, Medical University of Lodz, Lodz, Poland; 17 Institute for Clinical and Experimental Medicine – IKEM, Prague, Czech Republic; 18 Hospital Clínico Universitario de Valencia, INCLIVA, Universidad de Valencia, and CIBER Cardiovascular, Valencia, Spain; 19 Instituto de Cardiologia J. F. Cabral, Corrientes, Argentina; 20 Department of Cardiology, Herlev-Gentofte Hospital, University of Copenhagen, Herlev, Denmark; 21 Cardiovascular Department, ASST Papa Giovanni XXIII Hospital, Bergamo, Italy; 22 Department of Cardiology, University Medical Center Groningen, University of Groningen, Groningen, The Netherlands; 23 Division of Cardiology, Medical University of Graz, Graz, Austria; 24 Cardiology and Angiology, Medical Center – University of Freiburg, Faculty of Medicine, University of Freiburg, Freiburg, Germany; 25 Department of Cardiovascular Disease, Saint Luke’s Mid America Heart Institute, University of Missouri-Kansas City School of Medicine, Kansas City, MO USA

**Keywords:** Medical research, Drug discovery

## Abstract

In the STEP-HFpEF trial, semaglutide improved symptoms, physical limitations and exercise function and reduced body weight in patients with obesity phenotype of heart failure and preserved ejection fraction (HFpEF). This prespecified analysis examined the effects of semaglutide on dual primary endpoints (change in Kansas City Cardiomyopathy Questionnaire-Clinical Summary Score (KCCQ-CSS) and body weight) and confirmatory secondary endpoints (change in 6-minute walk distance (6MWD), hierarchical composite (death, HF events, change in KCCQ-CSS and 6MWD) and change in C-reactive protein (CRP)) across obesity classes I–III (body mass index (BMI) 30.0–34.9 kg m^−^^2^, 35.0–39.9 kg m^−^^2^ and ≥40 kg m^−^^2^) and according to body weight reduction with semaglutide after 52 weeks. Semaglutide consistently improved all outcomes across obesity categories (*P* value for treatment effects × BMI interactions = not significant for all). In semaglutide-treated patients, improvements in KCCQ-CSS, 6MWD and CRP were greater with larger body weight reduction (for example, 6.4-point (95% confidence interval (CI): 4.1, 8.8) and 14.4-m (95% CI: 5.5, 23.3) improvements in KCCQ-CSS and 6MWD for each 10% body weight reduction). In participants with obesity phenotype of HFpEF, semaglutide improved symptoms, physical limitations and exercise function and reduced inflammation and body weight across obesity categories. In semaglutide-treated patients, the magnitude of benefit was directly related to the extent of weight loss. Collectively, these data support semaglutide-mediated weight loss as a key treatment strategy in patients with obesity phenotype of HFpEF. ClinicalTrials.gov identifier: NCT04788511.

## Abstract (web summary)

In a prespecified secondary analysis of the STEP-HFpEF trial, semagludtide treatment was associated with improvements in KCCQ-CSS and body weight, as well as trial secondary endpoints, across the spectrum of obesity classes, with the magnitude of beneficial effects being proportional to the extent of weight loss.

## Main

The prevalence of heart failure with preserved ejection fraction (HFpEF) is increasing worldwide, and there are few effective treatments^1,2^. Approximately 60% of patients with HFpEF have the obesity phenotype^3^, which is a pathophysiologically distinct form of HFpEF characterized by greater symptom severity, poorer exercise capacity, more adverse hemodynamics and greater risk for HF hospitalization than those with HFpEF without obesity^3–10^. In the STEP-HFpEF trial, treatment with 2.4 mg of the glucagon-like peptide-1 receptor agonist semaglutide weekly produced substantial improvements in symptoms, physical limitations and exercise function and reduced inflammation and resulted in greater weight loss compared to placebo^11,12^.

However, it is not known if the observed effects of semaglutide in STEP-HFpEF vary by obesity class. Obesity is traditionally defined as body mass index (BMI) of 30 kg m^−^^2^ or greater, but, within this broad definition, there is substantial variation in the amount of excess adiposity. In the United States, approximately one-third of patients with the obesity phenotype of HFpEF have class III obesity, defined by BMI ≥40 kg m^−^^2^, whereas 40% of patients have class I obesity (BMI 30–34.9 kg m^−^^2^) (ref. ^3^). In cross-sectional studies, symptom severity, exercise limitations and hemodynamic abnormalities in the obesity phenotype of HFpEF worsen as BMI increases^6–8^, suggesting the possibility that beneficial effects from semaglutide could be mostly confined to individuals with HFpEF and very high BMI. Furthermore, it is unclear whether the magnitude of body weight reduction after treatment with semaglutide is related to the extent of clinical improvement in symptom severity, exercise function or systemic inflammation.

This prespecified analysis of STEP-HFpEF investigated the efficacy of semaglutide versus placebo in patients with HFpEF across the different classes of obesity, as it pertains to the primary endpoints (change in Kansas City Cardiomyopathy Questionnaire-Clinical Summary Score (KCCQ-CSS) and body weight) and confirmatory secondary endpoints (change in 6-minute walk distance (6MWD), hierarchical composite endpoint (comprising all-cause death, HF events, several thresholds of change in KCCQ-CSS and change in 6MWD ≥30 m) and change in C-reactive protein (CRP)), and it tested whether the degree of body weight reduction achieved on treatment with semaglutide was related to the improvements in the key trial endpoints.

## Results

### Patient characteristics

**Extended Data Fig. 1:**
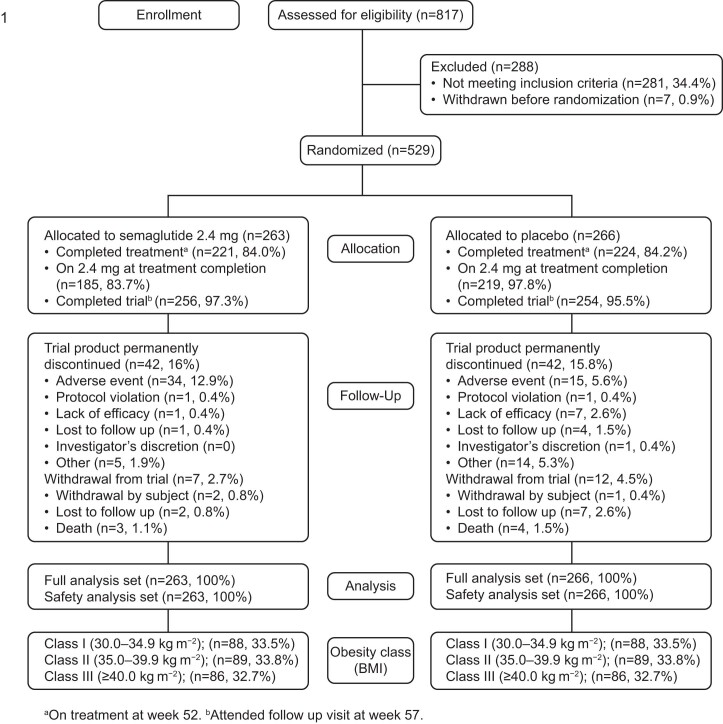
Participant flow diagram.

A total of 817 patients were screened, and, of this group, 529 fulfilled eligibility criteria and were enrolled and randomized between 19 March 2021 and 9 March 2022 (Extended Data Fig. 1). Among the 529 STEP-HFpEF participants, 263 and 266 were randomized to semaglutide versus placebo, respectively; the median BMI was 37.0 kg m^−^^2^ (33.7, 41.4) at baseline, 180 (34.0%) had class I obesity, 171 (32.3%) had class II obesity and 178 (33.7%) had class III obesity. Compared to patients who had less severe obesity, those with greater severity of obesity were more likely to be women and younger, with lower N-terminal pro-brain type natriuretic peptide (NTproBNP) levels but more severe impairments in HF symptoms, physical limitations and exercise function as reflected by lower KCCQ-CSS and 6MWD and higher New York Heart Association (NYHA) class and CRP levels (Table 1). No differences were observed in systolic blood pressure (SBP) or medical therapy for HF, except that patients with lower obesity class were more likely to be treated with sodium-glucose co-transporter-2 (SGLT2) inhibitors, and patients with higher obesity class were more likely to receive loop diuretics at higher dose. No differences were observed in the prevalence of hypertension, atrial fibrillation or sleep apnea by obesity class, but patients with increased severity of obesity were less likely to have history of coronary artery disease.

**Table 1:** Baseline characteristics of trial participants across obesity categories^a^

Characteristic	BMI <35 kg m^−^^2^ (*n* = 180)	BMI 35–<40 kg m^−^^2^ (*n* = 171)	BMI ≥40 kg m^−^^2^ (*n* = 178)	*P* value
Female, *n* (%)	91 (50.6)	88 (51.5)	118 (66.3)	0.0027
Age, years	72 (64, 78)	70 (63, 74)	67 (60, 73)	<0.0001
Ethnicity, *n* (%)^b^				0.8115
Hispanic or Latino	13 (7.2)	9 (5.3)	14 (7.9)	
Not Hispanic or Latino	167 (92.8)	162 (94.7)	164 (92.1)	
Race, *n* (%)^b^				0.1237
Black/African American	10 (5.6)	2 (1.2)	9 (5.1)	
White	170 (94.4)	169 (98.8)	168 (94.4)	
Other	0 (0.0)	0 (0.0)	1 (0.6)	
Body weight, kg	91.6 (84.1, 100.2)	105.8 (93.7, 117.5)	123.1 (110.0, 137.7)	<0.0001
BMI, kg m^−^^2c^	32.6 (31.3, 33.8)	37.1 (36.1, 38.4)	43.5 (41.3, 47.6)	^d^
Waist circumference, cm	110.0 (105.0, 116.8)	120.0 (113.0, 127.0)	129.0 (121.0, 141.0)	<0.0001
SBP, mmHg	132.0 (120.0, 141.5)	135.0 (122.0, 148.0)	132.0 (121.0, 140.0)	0.5912
NT-proBNP, pg ml^−1^	531.1 (278.7, 1083.8)	449.9 (205.5, 1058.8)	385.2 (181.0, 926.9)	0.0201
LVEF, %	56.0 (50.0, 60.0)	57.0 (50.0, 60.0)	58.0 (54.0, 61.0)	0.0206
LVEF stratification, *n* (%)				0.0928
45–49%^e^	33 (18.3)	31 (18.1)	21 (11.8)	
≥50%	147 (81.7)	140 (81.9)	157 (88.2)	
KCCQ-CSS score	61.7 (46.9, 76.0)	60.9 (46.9, 72.9)	51.6 (34.9–65.6)	<0.0001
6MWD, m	351.0 (260.5, 402.5)	340.0 (261.3, 400.0)	272.0 (207.6, 347.8)	<0.0001
CRP, mg L^−1^	2.6 (1.5, 5.9)	3.8 (2.0, 7.4)	5.2 (2.8, 10.2)	<0.0001
HF hospitalization within 1 year, *n* (%)	32 (17.8)	20 (11.7)	29 (16.3)	0.6926
Comorbidities at screening, *n* (%)				
Atrial fibrillation	97 (53.9)	88 (51.5)	90 (50.6)	0.5283
Hypertension	143 (79.4)	141 (82.5)	149 (83.7)	0.2950
Coronary heart disease	80 (44.4)	51 (29.8)	49 (27.5)	0.0007
Obstructive sleep apnea	24 (13.3)	15 (8.8)	27 (15.2)	0.6033
NYHA functional class, *n* (%)				0.0001
Class II	136 (75.6)	119 (69.6)	95 (53.4)	
Class III	44 (24.4)	51 (29.8)	83 (46.6)	
Class IV	0 (0.0)	1 (0.6)	0 (0.0)	
Concomitant medications, *n* (%)				
Diuretics	143 (79.4)	137 (80.1)	147 (82.6)	0.4520
Loop diuretics	109 (60.6)	95 (55.6)	125 (70.2)	0.0602
Loop diuretic dose (mg)^f^	40 (20, 40)	40 (20, 40)	40 (40, 80)	0.0002
Thiazides	33 (18.3)	31 (18.1)	26 (14.6)	0.3489
MRAs	56 (31.1)	58 (33.9)	70 (39.3)	0.1029
ACE/ARB (ARNI)	129 (71.7)	132 (77.2)	136 (76.4)	0.2992
ARNI	11 (6.1)	9 (5.3)	7 (3.9)	0.3492
Beta-blockers	150 (83.3)	132 (77.2)	136 (76.4)	0.1070
SGLT2i	10 (5.6)	7 (4.1)	2 (1.1)	0.0243

Percentages may not equal 100% due to rounding.

Two-sided *P* values for continuous variables are from a Jonckheere–Terpstra trend test, for binary variables from a Cochran–Armitage trend test and for multinomial variables from a Cochran–Mantel–Haenszel test.

^a^Data are median (Q1, Q3) unless otherwise stated and are from the full analysis set.

^b^Race and ethnic group were reported by the investigator.

^c^BMI is the weight in kilograms divided by the square of the height in meters.

^d^Not relevant.

^e^Includes one participant with LVEF of 33%.

^f^Reported in furosemide equivalents per day.

ACEI, angiotensin-converting enzyme inhibitor; ARB, angiotensin II receptor blocker; ARNI, angiotensin receptor-neprilysin inhibitor; MRA, mineralocorticoid receptor antagonist; Q, quartile; SGLT2i, sodium-glucose co-transporter-2 inhibitor.

In regression analyses, increase in BMI was associated with lower KCCQ-CSS and 6MWD and higher CRP at the time of baseline assessment, after adjusting for age, sex, NYHA class, history of atrial fibrillation and history of coronary disease (Supplementary Table 1).

### Treatment effects by baseline obesity class

**Fig. 1:**
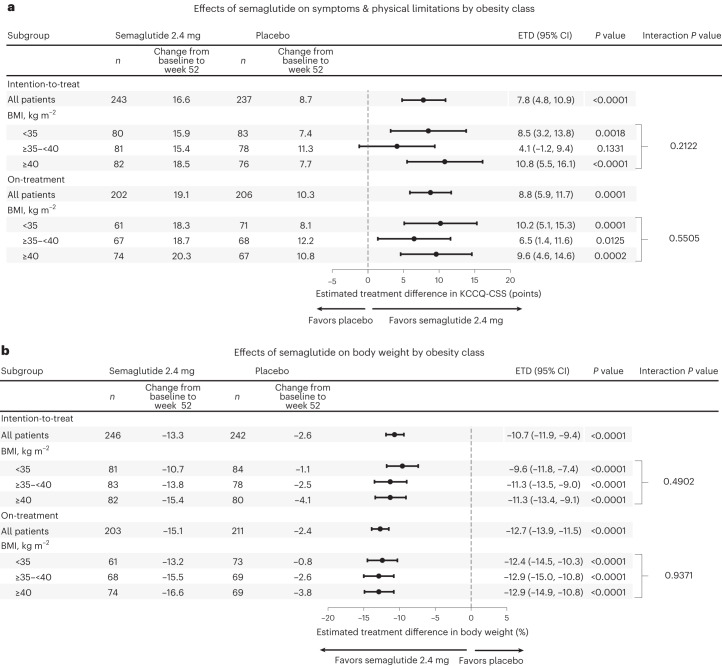
Effects of semaglutide compared to placebo across different obesity classes on HF symptoms, physical limitations and body weight. **a**,**b**, There was no evidence of heterogeneity in the effects of semaglutide compared to placebo on the dual primary endpoints of KCCQ-CSS (**a**) or body weight (**b**). Data are point estimates and 95% CIs. Analyses using the intention-to-treat principle employ an F-test for interaction and a Wald test for treatment effect within BMI subgroups, with 1,000 imputations using Rubin’s rule. Analyses using on-treatment data employ an F-test for interaction and a *t*-test for treatment effect within BMI subgroups. *P* values are two-sided. ETD, estimated treatment difference.

**Fig. 2:**
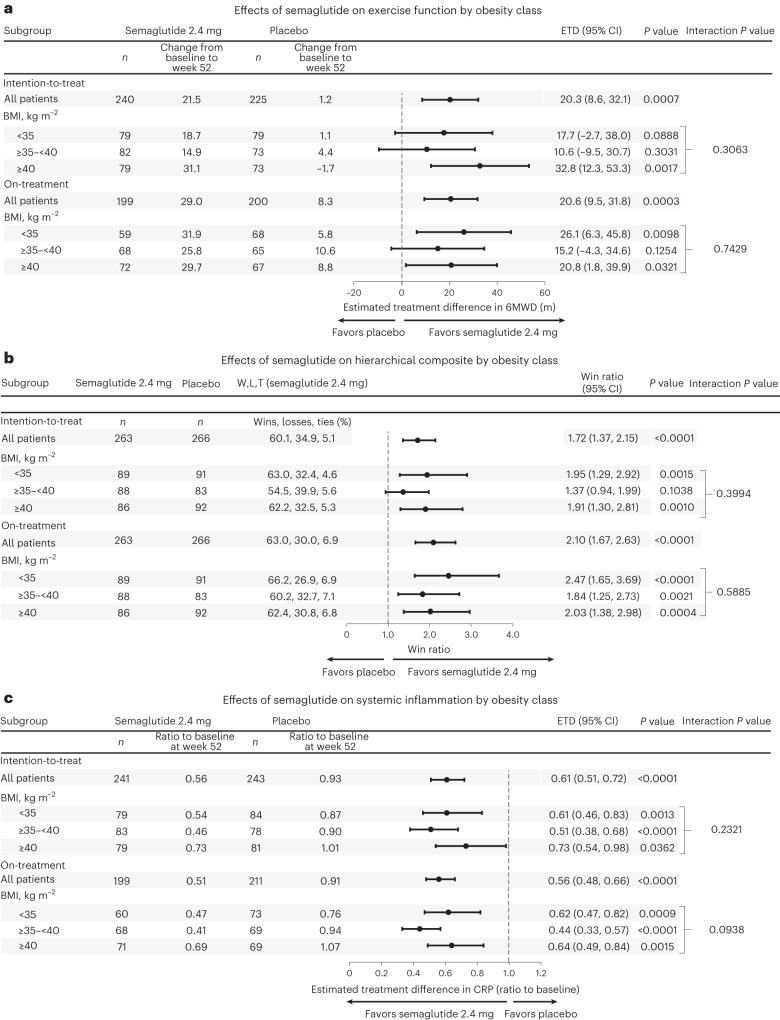
Effects of semaglutide compared to placebo across different obesity classes on exercise function, hierarchical composite endpoint and systemic inflammation. **a**,**b**, There was no evidence of heterogeneity in the effects of semaglutide compared to placebo on the confirmatory secondary endpoints of exercise function assessed by 6MWD (**a**), the hierarchical composite endpoint (**b**) or systemic inflammation assessed by CRP levels. Data are point estimates and 95% CIs. Analyses using the intention-to-treat principle employ an F-test (**a**,**c**) or Cohran’s Q-test (**b**) for interaction and a Wald test for treatment effect within BMI subgroups, with 1,000 imputations using Rubin’s rule. Analyses using on-treatment data employ an F-test for interaction and a *t*-test for treatment effect within BMI subgroups (**a**,**c**) or Cohran’s Q-test (**b**) for interaction and a Wald test for treatment effect within BMI subgroups. *P* values are two-sided. Other abbreviations as in Fig. 1.

As compared to placebo, treatment with semaglutide improved KCCQ-CSS and reduced body weight across all obesity categories (Fig. 1). Semaglutide also improved 6MWD, resulted in a greater number of wins versus placebo for the composite hierarchical endpoint and reduced systemic inflammation assessed by CRP in each obesity class, with no heterogeneity of treatment benefits (Fig. 2). These findings were observed in both the intention-to-treat analyses and the on-treatment analyses.

### Semaglutide effects and weight change

**Fig. 3:**
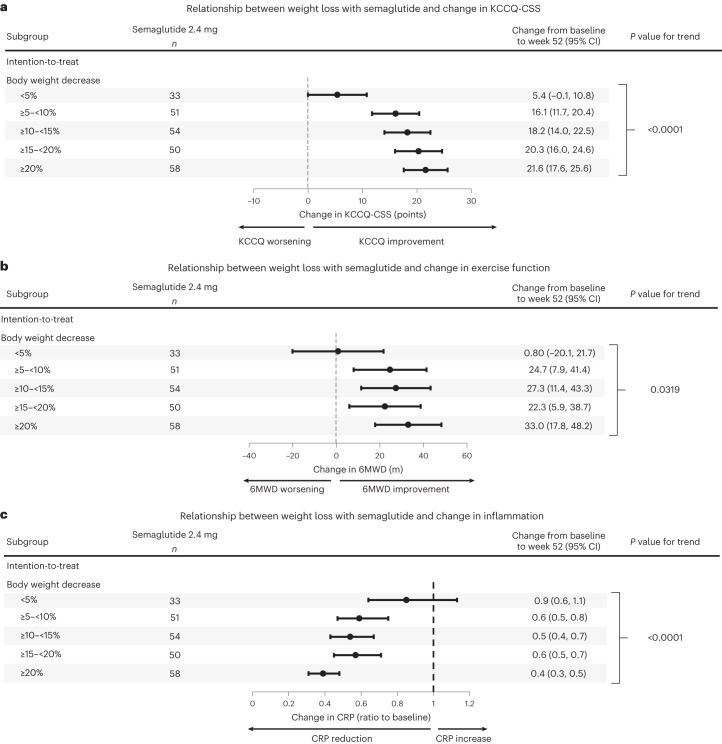
Relationship between the magnitude of body weight reduction on semaglutide and primary and confirmatory secondary endpoints. **a**–**c**, Greater body weight reduction with semaglutide was associated with greater improvements in HF symptoms and physical limitations assessed by the KCCQ-CSS (**a**), exercise function assessed by the 6MWD (**b**) and greater reduction in systemic inflammation assessed by CRP levels (**c**). Data are point estimates and 95% CIs. Analyses use the intention-to-treat principle; tests for trend are based on an F-test. *P* values are two-sided. Abbreviations as in Figs. 1 and 2.

**Extended Data Fig. 2:**
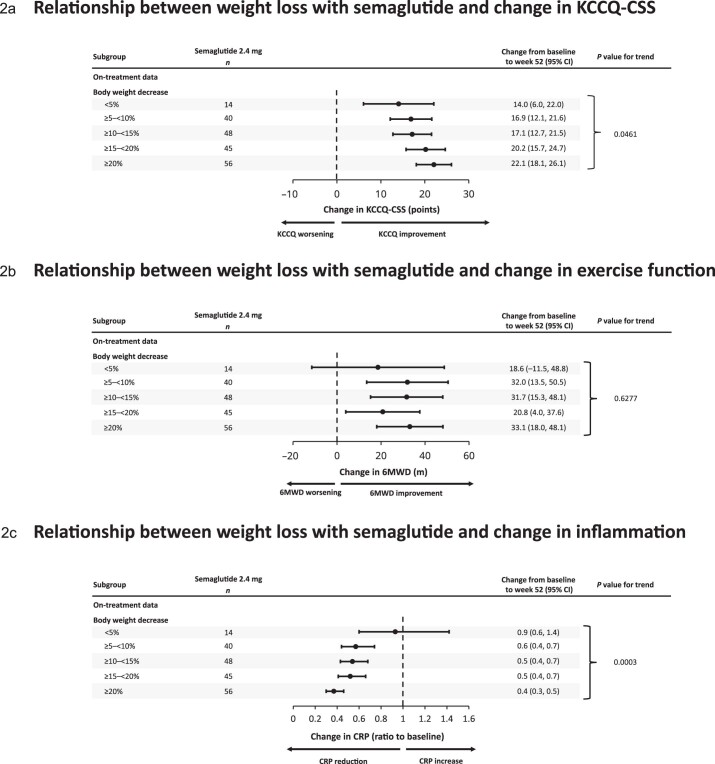
**Relationship between the magnitude of body weight reduction on semaglutide with change in KCCQ-CSS (a); 6MWD (b); and ratio of CRP to baseline (c) in the on-treatment (per protocol) analysis. Data are point estimates and 95% CIs. Tests for trend are based on an F-test;** ***P*** **values are two-sided**.

Among patients who were treated with semaglutide and had a recorded body weight at week 52, body weight reduction was <5% in 33 (13.4%) participants, 5–<10% in 51 (20.7%) participants, 10–<15% in 54 (22.0%) participants, 15–<20% in 50 (20.3%) participants and >20% in 58 (23.6%) participants. Increased degree of body weight reduction was associated with increased magnitude of improvements in KCCQ-CSS and 6MWD and reduction in CRP. These dose–response relationships between the amount of weight loss and treatment benefits were observed when body weight change was analyzed both as an ordinal (Fig. 3) and as a continuous variable, after adjusting for age, sex, NYHA class, history of atrial fibrillation and coronary artery disease, baseline CRP and NTproBNP at baseline (Table 2). Results were consistent between the intention-to-treat and on-treatment analyses, except for 6MWD where dose–response relationship was observed in the intention-to-treat, but not in the on-treatment, analysis (Extended Data Fig. 2).

**Table 2:** Regression analysis of changes in body weight on semaglutide to efficacy outcomes

	Predicted change per 10% decrease in body weight Model 1		Predicted change per 10% decrease in body weight Model 2	
Endpoint change at 52 weeks	Slope (95% CI)^a^	*P*	Slope (95% CI)^a^	*P*
KCCQ-CSS (points)	5.9 (3.6, 8.3)	<0.0001	6.4 (4.1, 8.8)	<0.0001
6MWD (m)	13.2 (4.4, 22.0)	0.0033	14.4 (5.5, 23.3)	0.0016
CRP (ratio)	0.75 (0.65, 0.86)	<0.0001	0.72 (0.63, 0.84)	<0.0001

Results are shown for the intention-to-treat analysis. Data are point estimates and 95% CIs, computed using multivariable regression analyses. *P* values are two-sided.

Model 1 is adjusted for baseline weight and baseline endpoint (baseline KCCQ-CSS, 6MWD or logarithm to CRP).

Model 2 is adjusted for baseline values of weight, respective endpoint, age, sex, history of atrial fibrillation, history of coronary artery disease, NYHA class, logarithm to CRP and logarithm to NTproBNP.

^a^Change per 10% decrease in body weight on treatment with semaglutide.

Based on the linear regression slopes (Table 2), each 10% reduction in body weight with semaglutide was associated with a 6.4-point (95% confidence interval (CI): 4.1, 8.8) increase in KCCQ-CSS, a 14.4-m (95% CI: 5.5, 23.3) increase in 6MWD and a 28% (95% CI: 16, 37) decrease in CRP, after adjusting for baseline age, sex, body weight, endpoint value, NYHA class, coronary artery disease, atrial fibrillation, CRP (log-transformed) and NTproBNP (log-transformed).

### Safety and tolerability

There were fewer serious adverse events reported among participants randomized to semaglutide versus placebo within each obesity class, with no evidence of heterogeneity in safety or tolerability (Table 3). A similar (and small) number of patients discontinued study medication due to serious adverse events in the semaglutide and placebo groups. The number of deaths in the semaglutide and placebo groups, respectively, were 2 and 0 in obesity class I, 0 and 2 in obesity class II and 1 and 2 in obesity class III.

**Table 3:** Adverse events

	Adverse event rate per 100 patient years					
	Class I obesity (30–34.9 kg m^−^^2^)		Class II obesity (35–39.9 kg m^−^^2^)		Class III obesity (≥40 kg m^−^^2^)	
	Placebo (*n* = 91)	Semaglutide (*n* = 89)	Placebo (*n* = 83)	Semaglutide (*n* = 88)	Placebo (*n* = 92)	Semaglutide (*n* = 86)
Serious adverse events	53.7	32.2	39.0	18.4	56.7	20.4
Deaths	0.0	2.5	3.5	0.0	2.2	1.1
Category of serious adverse event						
Cardiac disorders	19.7	7.4	9.5	1.2	18.9	1.1
Infections and infestations	3.3	3.7	13.0	2.3	8.9	0.0
Gastrointestinal disorders	3.3	2.5	0.0	4.6	5.6	3.4
Nervous system disorders	3.3	6.2	3.5	1.2	1.1	2.3
Renal and urinary disorders	1.1	3.7	1.2	1.2	4.4	3.4
Respiratory, thoracic and mediastinal	6.6	0.0	2.4	0.0	3.3	0.0
Musculoskeletal and connective tissue	1.1	0.0	1.2	3.5	3.3	2.3
Injury, poisoning and procedural	3.3	0.0	2.4	2.3	0.0	2.3
Metabolism and nutrition disorders	2.2	0.0	1.2	2.3	1.1	1.1
Hepatobiliary disorders	2.2	3.7	0.0	0.0	0.0	1.1
General disorders and administration site	0.0	0.0	1.2	0.0	2.2	1.1
Neoplasms benign, malignant and unspecified	1.1	0.0	1.2	0.0	1.1	1.1
Serious adverse event leading to discontinuation	4.4	5.0	1.2	1.2	2.2	2.3

## Discussion

In this prespecified analysis from the STEP-HFpEF trial, semaglutide as compared to placebo improved HF-related symptoms, physical limitations and exercise function and reduced body weight and inflammation across the spectrum of obesity categories. Furthermore, in patients treated with semaglutide, increased degree of weight loss was associated with increased magnitude of improvements in symptoms, physical limitations and inflammation, even after adjusting for relevant baseline characteristics that might influence treatment response, including age, sex and baseline body weight. These data demonstrate that the effects of semaglutide-induced weight loss are not restricted to individuals with very high BMI but apply across the entire spectrum of obesity. In addition, the relationships between the magnitude of weight reduction and clinical efficacy provide mechanistic evidence supporting the importance of weight reduction as an effective treatment for patients with the obesity phenotype of HFpEF.

Patients with the obesity phenotype of HFpEF display distinct pathophysiologic characteristics compared to patients with other phenotypes of HFpEF, including greater volume expansion, higher cardiac filling pressures, more severe right-sided HF and increases in epicardial fat that amplify external constraint on the heart^6^. It has been shown that patients with the obesity HFpEF phenotype are younger and have lower natriuretic peptide levels^3,6,7^ but present with higher NYHA class^4,7^, greater symptom severity, poorer exercise capacity^7,8^ and greater systemic inflammation than patients without obesity^7^. These relationships with obesity severity in patients with HFpEF were again observed in the present analysis, supporting the validity and generalizability of these data from the STEP-HFpEF trial.

Previous studies showed direct linear relationships between body weight and symptom severity, exercise limitation and hemodynamic abnormalities in patients with the obesity phenotype of HFpEF^6–8^. These relationships might support a hypothesis that only those individuals with HFpEF and the most severe obesity phenotypes would benefit from weight loss treatments. However, in this analysis, we observed similar treatment benefits of semaglutide for all primary and confirmatory secondary endpoints across the spectrum of obesity categories. These findings have important clinical implications, as approximately 40% of patients with the obesity phenotype of HFpEF have only mild obesity (class I), and the present analyses indicate that these patients benefit just as much as patients with more severe obesity^3^. The relationship observed between reductions in body weight and improvements in symptoms and physical limitations supports the hypothesis that the obesity phenotype of HFpEF is, in large part, a consequence of increased adiposity and its many sequelae, although there are also likely non-obesity-related contributors to the pathophysiology.

Weight loss in patients with obesity but no HF is associated with effects that would be expected to reduce symptom severity and improve exercise function in patients with HFpEF, including reversal of hypertrophic chamber remodeling, improvement in ventricular mechanics and reductions in hemodynamic congestion^13,14^. The present findings are supported by the results of the SECRET trial, which showed that non-pharmacologic body weight reduction achieved through caloric restriction improved exercise capacity in patients with the obesity phenotype of HFpEF^15^. The present study directly relates the degree of pharmacologically mediated weight loss with the magnitude of clinical benefits observed across the broad range of outcomes, including symptoms and physical limitations (KCCQ-CSS), exercise function (6MWD) and inflammation (CRP). These benefits are not simply ascribable to mechanical effects of body weight reduction, as the STEP-HFpEF trial also showed that semaglutide reduced NTproBNP levels compared to placebo, consistent with a direct benefit on hemodynamic congestion^11^.

The findings of this study should be considered in the context of several potential limitations. Most participants in STEP-HFpEF were White, and individuals with diabetes were excluded by design, which may affect the generalizability to non-White populations and people with diabetes. A separate, ongoing trial is evaluating the effects of semaglutide in people with the obesity phenotype of HFpEF and type 2 diabetes^12^. The STEP-HFpEF trial was designed to evaluate the effects of treatment on symptoms and physical limitations, exercise function and inflammation, along with body weight, and was, therefore, not powered to assess clinical endpoints such as HF hospitalizations. Power is reduced by focusing on obesity class subgroups as compared to the main analysis, but the findings were consistent across obesity categories with no evidence for heterogeneity of treatment effects. The 52-week duration of treatment was relatively short, and whether the observed effects might have persisted (or become more amplified) with longer evaluation is not known. Use of SGLT2 inhibitors was low in STEP-HFpEF, as patients with diabetes were excluded, and these agents were not yet approved for treatment of HFpEF during the trial conduct. Although semaglutide and SGLT2 inhibitors have complementary and non-overlapping mechanisms of action, the present study cannot determine whether background therapy with SGLT2 inhibitors might have influenced the treatment benefits observed, which is an important question for future trials. Further insight into the effects of semaglutide in patients who receive background SGLT2 inhibitors will be provided by the STEP-HFpEF DM trial, which includes a greater proportion (32%) of patients taking these agents^12^. BMI is a crude measure of adiposity that does not assess body composition or quantity of visceral fat, which has more deleterious effects in HFpEF^6,16^, limiting insight on the effect of semaglutide on visceral fat loss and its association with improvements in KCCQ-CSS and 6MWD outcomes.

In the STEP-HFpEF trial of participants with the obesity phenotype of HFpEF, semaglutide improved symptoms, physical limitations and exercise function and reduced inflammation and body weight across the spectrum of obesity categories. In semaglutide-treated patients, the magnitude of benefit was directly related to the extent of weight loss. Collectively, these data support semaglutide-mediated weight loss as a key treatment strategy in patients with the obesity phenotype of HFpEF.

## Methods

### Study design

STEP-HFpEF (NCT04788511) was a randomized, international, double-blind, placebo-controlled trial that examined the efficacy and safety of semaglutide 2.4 mg once weekly compared to placebo in patients with the obesity phenotype of HFpEF without diabetes^11^. The study design and the primary results were previously published^11,12^. Institutional review board ethics approval was obtained at each study site, and all patients provided informed consent to participate in the trial.

### Study patients

Eligible participants were randomized 1:1 to semaglutide 2.4 mg subcutaneously or matching placebo once weekly in addition to standard of care for 52 weeks^12^. For all participants, frequent physical activity of moderate intensity (as tolerated in HFpEF) and limited consumption of salt, red meat, saturated or trans fats, sweets and sugar-sweetened beverages, with restricted calorie intake (goal, 500 kcal deficit per day) were recommended. Smoking cessation was supported, and alcohol consumption was recommended to be limited. Patients were eligible if they had left ventricular ejection fraction (LVEF) ≥45%, NYHA functional class II–IV, BMI ≥30 kg m^−^^2^, KCCQ-CSS <90 points and objective evidence of HF based on at least one of the following criteria: (1) elevated left ventricular filling pressures (pulmonary artery wedge pressure or left ventricular end-diastolic pressure ≥15 mmHg at rest or ≥25 mmHg with exercise documented during catheterization or pulmonary artery diastolic pressure measured by implantable monitor ≥15 mmHg, assessed invasively); (2) elevated natriuretic peptide levels (with thresholds stratified based on BMI: ≥220 pg ml^−1^ for patients with BMI <35.0 and sinus rhythm; ≥660 pg ml^−1^ for patients with BMI <35.0 and persistent/permanent atrial fibrillation; ≥125 pg ml^−1^ for patients with BMI ≥35.0 and sinus rhythm; or ≥375 pg ml^−1^ for patients with BMI ≥35.0 and persistent/permanent atrial fibrillation, together with echocardiographic abnormalities (at least one of the following: (i) septal é <7 cm s^−1^ or lateral é < 10 cm s^−1^ or average E/é ≥15; (ii) pulmonary artery systolic pressure >35 mmHg; (iii) left atrial enlargement defined by local laboratory; and (iv) left ventricular hypertrophy with septal thickness or posterior wall thickness ≥1.2 cm)); or (3) hospitalization for HF in the preceding 12 months plus requirement for ongoing diuretics and/or echocardiographic abnormalities (as defined above). Key exclusion criteria were previous or planned bariatric surgery, self-reported change in body weight >11 pounds (5 kg) within 90 d before randomization or SBP >160 mmHg at screening. Patients were excluded from the trial if they had a HbA1c level ≥6.5% or prior medical history of diabetes, because clinical characteristics and response to semaglutide may differ in patients with diabetes. A sister trial (STEP-HFpEF DM) is evaluating the effects of semaglutide in patients with obesity phenotype of HFpEF and diabetes (NCT04916470). The STEP-HFpEF trial was sponsored by Novo Nordisk.

### BMI and weight changes

BMI was calculated as body weight in kilograms divided by height in meters squared based on measurements at baseline before randomization. Patients were stratified into BMI categories as obesity class I (BMI 30–34.9 kg m^−^^2^), class II (BMI 35–39.9 kg m^−^^2^) or class III (BMI ≥40 kg m^−^^2^). Relative changes in body weight were expressed as the difference in body weight between baseline and 52 weeks divided by baseline body weight calculated as percentage.

### Outcomes

The dual primary endpoints of STEP-HFpEF were change in KCCQ-CSS and percent change in body weight from baseline to 52 weeks^11,12^. Confirmatory secondary endpoints included exercise function assessed by change in 6MWD, overall clinical benefit assessed using a hierarchical composite endpoint (all-cause death, HF events, several thresholds of change in KCCQ-CSS from baseline to 52 weeks and change in 6MWD ≥30 m) and change in CRP from baseline to 52 weeks. All serious adverse events and adverse events leading to premature treatment discontinuation were reported to evaluate safety and tolerability.

### Statistical analysis

Baseline characteristics were evaluated according to BMI groups (30–<35, 35–<40 and ≥40 kg m^−^^2^), and tests for trend were performed across these groups. Efficacy endpoints for semaglutide compared to placebo, stratified by obesity class at baseline, were assessed using both the full analysis set (all randomized participants according to the intention-to-treat principle, regardless of treatment discontinuation) and the on-treatment data (including only patients receiving allocated study medication). Weight loss ‘dose–effect’ analyses were performed according to the magnitude of body weight change during the trial confined to the semaglutide group, because the primary objective was to examine the effects of body weight change related to semaglutide treatment rather than spontaneous or other lifestyle-related weight changes (as in the placebo group), using both intention-to-treat (primary) and on-treatment approaches. Subgroup analyses for continuous endpoints in the intention-to-treat were performed using 1,000 multiple imputations using analysis of covariance models, with treatment by BMI groups adjusted for the relevant continuous baseline variable^12^. Estimates from the multiple imputations were derived using Rubin’s rule. Subgroups analyses of the hierarchical composite endpoint (win ratio) were performed stratified by the obesity category, based on direct comparisons of each participant randomized to semaglutide versus each participant randomized to placebo within each BMI subgroup. For each of these participant pairs, a ‘treatment winner’ based on similar observation time was declared based on the endpoint hierarchy (as previously reported^11,12^). The win ratio (that is, the proportion of winners randomized to semaglutide divided by the winners randomized to placebo) was estimated independently within each BMI subgroup using 1,000 imputations. Test for equality of the BMI groups for the win ratio was performed using Cohranʼs Q-test. Subgroup analyses for continuous endpoints in relation to the secondary hypothetical estimand (on treatment with trial product) were performed using a mixed model with treatment by BMI group adjusted for the relevant continuous baseline variable, all nested within visit, and treatment by BMI groups was evaluated at week 52 using on-treatment data. The hierarchical endpoint was analyzed using prediction (single-imputed) from a mixed model using on-treatment data for each of the components and analyzed stratified as described above. All imputations for the win ratio were pertinent only to KCCQ-CSS and 6MWD, where all-cause death and HF events differed between intention-to-treat and on-treatment approaches due to the collection of events in these two trial periods. Multivariable regression analyses were performed to determine independent relationships between baseline BMI and baseline outcome measures before treatment after adjusting a priori for baseline characteristics that might confound interpretation (age, sex, NYHA class, history of atrial fibrillation and history of coronary artery disease). Multivariable linear regression was also performed to determine relationships between change in body weight and changes in study outcomes with semaglutide unadjusted and after adjusting (a priori) for age, sex, NYHA functional class, history of coronary artery disease, history of atrial fibrillation, baseline CRP and baseline NTproBNP levels. Both unadjusted and adjusted analyses included baseline body weight and relevant continuous baseline variables (for example, baseline KCCQ-CSS, 6MWD or CRP) as covariates. Change in body weight was analyzed both as a continuous variable (% change from baseline) and as an ordinal variable, including the following weight loss categories from baseline to 52 weeks: <5%, 5–<10%, 10–<15%, 15–<20% and ≥20%. A test for linearity was employed for the categorial weight change analyses. All results from statistical analyses are presented with two-sided *P* values and 95% CIs. Safety endpoints were analyzed using the safety analysis set (all randomized participants exposed to at least one dose of randomized treatment). Further details on the estimands, including specification of intention-to-treat and on-treatment data, statistical analyses and imputation methods to account for missing data, were previously published^12^. The primary estimand quantified the average change from baseline to 52 weeks in KCCQ-CSS and body weight of semaglutide 2.4 mg once weekly relative to placebo, both added to standard of care, in all randomized participants regardless of adherence to randomized treatment. *P* values less than 5% were considered significant, and no adjustment for multiplicity was performed.

### Reporting summary

Further information on research design is available in the Nature Portfolio Reporting Summary linked to this article.

## Online content

Any methods, additional references, Nature Portfolio reporting summaries, source data, extended data, supplementary information, acknowledgements, peer review information; details of author contributions and competing interests; and statements of data and code availability are available at 10.1038/s41591-023-02526-x.

## Supplementary information

Supplementary Information Supplementary Table 1

Reporting Summary

## Data Availability

Data will be shared with bona fide researchers submitting a research proposal approved by the independent review board. Instructions for submitting proposals can be found at https://www.novonordisk-trials.com/. Data will be made available after research completion and approval of the product and product use in the European Union and the United States. Individual participant data will be shared in datasets in a de-identified/anonymized format.

## References

[1] Redfield, M. M. & Borlaug, B. A. Heart failure with preserved ejection fraction: a review. *JAMA* **329**, 827–838 (2023). 36917048 10.1001/jama.2023.2020

[2] Borlaug, B. A., Sharma, K., Shah, S. J. & Ho, J. E. Heart failure with preserved ejection fraction: JACC Scientific Statement. *J. Am. Coll. Cardiol.* **81**, 1810–1834 (2023). 37137592 10.1016/j.jacc.2023.01.049

[3] Morgen, C. S. et al. Obesity, cardiorenal comorbidities and risk of hospitalization in patients with heart failure with preserved ejection fraction. *Mayo Clin. Proc*. 10.1016/j.mayocp.2023.07.008 (2023). 10.1016/j.mayocp.2023.07.008 37565948

[4] Dalos, D. et al. Functional status, pulmonary artery pressure, and clinical outcomes in heart failure with preserved ejection fraction. *J. Am. Coll. Cardiol.* **68**, 189–199 (2016). 27386773 10.1016/j.jacc.2016.04.052

[5] Kitzman, D. W. & Shah, S. J. The HFpEF obesity phenotype: the elephant in the room. *J. Am. Coll. Cardiol.* **68**, 200–203 (2016). 27386774 10.1016/j.jacc.2016.05.019

[6] Obokata, M., Reddy, Y. N. V., Pislaru, S. V., Melenovsky, V. & Borlaug, B. A. Evidence supporting the existence of a distinct obese phenotype of heart failure with preserved ejection fraction. *Circulation* **136**, 6–19 (2017). 28381470 10.1161/CIRCULATIONAHA.116.026807 PMC5501170

[7] Reddy, Y. N. V. et al. Characterization of the obese phenotype of heart failure with preserved ejection fraction: a RELAX trial ancillary study. *Mayo Clin. Proc.* **94**, 1199–1209 (2019). 31272568 10.1016/j.mayocp.2018.11.037

[8] Reddy, Y. N. V. et al. Quality of life in heart failure with preserved ejection fraction: importance of obesity, functional capacity, and physical inactivity. *Eur. J. Heart Fail.* **22**, 1009–1018 (2020). 32150314 10.1002/ejhf.1788

[9] Adamson, C. et al. Dapagliflozin for heart failure according to body mass index: the DELIVER trial. *Eur. Heart J.* **43**, 4406–4417 (2022). 36029309 10.1093/eurheartj/ehac481 PMC9622300

[10] Borlaug, B. A. et al. Obesity and heart failure with preserved ejection fraction: new insights and pathophysiological targets. *Cardiovasc. Res.* **118**, 3434–3450 (2023). 35880317 10.1093/cvr/cvac120 PMC10202444

[11] Kosiborod, M. N. et al. Once weekly semaglutide in heart failure with preserved ejection fraction and obesity. *N. Engl. J. Med*. (in the press). 10.1056/NEJMc2312296 38118037

[12] Kosiborod, M. N. et al. Design and baseline characteristics of STEP-HFpEF program evaluating semaglutide in patients with obesity HFpEF phenotype. *JACC Heart Fail.* **11**, 1000–1010 (2023). 37294245 10.1016/j.jchf.2023.05.010

[13] Sorimachi, H. et al. Long-term changes in cardiac structure and function following bariatric surgery. *J. Am. Coll. Cardiol.* **80**, 1501–1512 (2022). 36229085 10.1016/j.jacc.2022.08.738 PMC9926898

[14] Reddy, Y. N. V. et al. Hemodynamic effects of weight loss in obesity: a systematic review and meta-analysis. *JACC Heart Fail.* **7**, 678–687 (2019). 31302042 10.1016/j.jchf.2019.04.019 PMC6677608

[15] Kitzman, D. W. et al. Effect of caloric restriction or aerobic exercise training on peak oxygen consumption and quality of life in obese older patients with heart failure with preserved ejection fraction: a randomized clinical trial. *JAMA* **315**, 36–46 (2016). 26746456 10.1001/jama.2015.17346 PMC4787295

[16] Sorimachi, H. et al. Pathophysiologic importance of visceral adipose tissue in women with heart failure and preserved ejection fraction. *Eur. Heart J.* **42**, 1595–1605 (2021). 33227126 10.1093/eurheartj/ehaa823 PMC8060057

