# Supplementary material for: Semaglutide in HFpEF across obesity class and by body weight reduction: a prespecified analysis of the STEP-HFpEF trial
Source: Nat Med. 2023 Aug 27;29(9):2358–65. doi: 10.1038/s41591-023-02526-x (PMC10504076; doi:10.1038/s41591-023-02526-x)
Supplement: Supplementary file 1 — Supplementary Table 1 [file 41591_2023_2526_MOESM1_ESM.pdf]

nature medicine

Article https://doi.org/10.1038/s41591-023-02526-x

Semaglutide in HFpEF across obesity class and by body weight reduction: a prespecified analysis of the STEP-HFpEF trial

In the format provided by the authors and unedited

Contents page

List of clinical sites in the STEP-HFpEF trial 2

Supplementary Table 1: Regression Analysis of Baseline BMI (Continuous) and Baseline Endpoint Assessments (Prior to Treatment) 6

List of clinical sites in the STEP-HFpEF trial

Site number	Country	Organization
101	Argentina	Sanatorio Britanico S.A.
102	Argentina	Centro de Investigación y Prevención Cardiovascular
103	Argentina	Instituto de Cardiología de Corrientes
104	Argentina	Consultorio Integral de Atención al Diabético
105	Argentina	CEMEDIC
106	Argentina	Cardiología Palermo
150	Australia	Geelong Cardiology Research Unit
151	Australia	Concord Repatriation General Hospital - Cardiology
152	Australia	Liverpool Hospital
153	Australia	The Canberra Hospital_Garran
154	Australia	Flinders Medical Centre
201	Canada	Dr James Cha
202	Canada	Cambridge Cardiac Care Centre
203	Canada	Institut Universitaire de Cardiologie et de Pneumologie. de Quebec - Universite Laval
204	Canada	Heart Health Institute Research, Inc.
205	Canada	Dr. Louis Yao
206	Canada	Oakville Cardiovascular Research LP
209	Canada	Partnrs Adv Cardio Eval (PACE)
250	Czech Republic	Vseobecna fakultni nemocnice a 1 LF UK v Praze
251	Czech Republic	IKEM
252	Czech Republic	Nemocnice Jihlava Kardiologie
254	Czech Republic	Poliklinika Holešovice VISIONARY - Medicon Pharm s.r.o.
255	Czech Republic	Nemocnice Slany Kardiologie
300	Germany	Hausaerztlich-Kardiologisches MVZ Am Felsenkeller GmbH
301	Germany	Appel
302	Germany	MVZ CCB Frankfurt Und Main-Taunus GbR
303	Germany	Universitatsklinikum Wurzburg AöR
304	Germany	Medical Center - University Of Freiburg

305	Germany	Charite Universitatsmedizin Berlin KöR
306	Germany	Zentrum fuer klinische Studien Suedbrandenburg GmbH
350	Denmark	Herlev og Gentofte Hospital
351	Denmark	Kardiologisk Odense & Svendborg
352	Denmark	Aarhus Universitetshospital, Skejby Hjertesygdomme
401	Hungary	Semmelweis Egyetem Városmajori Szív- és Érgyógyászat
402	Hungary	Zala Megyei Szent Rafael Kórház
403	Hungary	Borsod-Abaúj-Zemplén Megyei Központi Kórház
404	Hungary	Jahn Ferenc Dél-pesti Kórház és Rendelőintézet
405	Hungary	Szent Margit Rendelőintézet Nonprofit Kft.
407	Hungary	Semmelweis Egyetem Szent Rókus Klinikai Tömb
408	Hungary	Lausmed Kft.
409	Hungary	Selye János Kórház
451	Israel	Cardio Vascular Research Center Sourasky MC
452	Israel	Sheba Medica Center - Clinical Research Unit
453	Israel	Heart Failure Unit, Rabin Medical Center - Beilinson Campus
454	Israel	Hadassah MC - Cardio
455	Israel	Cardiology department, Western Galilee Medical Center
500	Netherlands	UMC Groningen
501	Netherlands	Gelre Ziekenhuizen Apeldoorn
502	Netherlands	Medisch Centrum Leeuwarden
503	Netherlands	Saxenburgh Medisch Centrum
504	Netherlands	Rode Kruis Ziekenhuis Beverwijk
505	Netherlands	Bravis Ziekenhuis
550	Poland	Uniwersytecki Szpital Kliniczny Im Wojskowej Akademii Medycznej Centralny Szpital Weteranow
551	Poland	I Katedra i Klinika Kardiologii WUM SPCSK
552	Poland	Ind. Prak. Lek. w dziedz. Kardiologii lek. med. K. Cymerman
553	Poland	Uniwersytecki Szpital Kliniczny w Bialymstoku K. Kardio
554	Poland	Pro Familia Altera Sp. z o.o.
555	Poland	Malopolskie Centrum Sercowo-Naczyniowe
601	Spain	Hospital Clínico Universitario de Valencia

602	Spain	Complejo Hospitalario Universitario de Santiago
603	Spain	Hospital Universitario La Zarzuela
650	United Kingdom	Glasgow Clinical Research Facility
651	United Kingdom	Manchester Royal Infirmary_Manchester_0
652	United Kingdom	Queen Elizabeth University Hospital
653	United Kingdom	Ninewells Hospital
654	United Kingdom	Wycombe General Hospital
655	United Kingdom	St. Richards Hospital
656	United Kingdom	Great Western Hospital
657	United Kingdom	Southmead Hospital
658	United Kingdom	University Hospital Aintree
701	United States	Chicago Medical Research LLC
703	United States	Saint Luke's Hospital of Kansas City
704	United States	Eastern Shore Rsrch Inst, LLC
705	United States	St Louis Heart & Vascular, P.C.
706	United States	NY Presbyt Hosp-W Cornell Med
707	United States	Wake Forest School of Medicine
708	United States	Northwest Heart Clinical Research, LLC
709	United States	St Francis Hospital Lindner Research Center
710	United States	CHI Health Clinic Cardiology (CUMC - Bergan Mercy)
711	United States	The Lindner Center for Research and Education at The Christ Hospital
712	United States	Keck Medical Center of USC - Outpatient Clinic
714	United States	Virginia Heart
717	United States	John Hopkins Hospital
718	United States	Univ of Mississippi Med Ctr
719	United States	Cotton-O'Neil Heart Center
720	United States	Baptist Heart Specialists_Jacksonville
721	United States	Hospital of the University of Pennsylvania
722	United States	Northwestern University_Chicago_0
723	United States	Ascension St. Vincent Medical Group
730	United States	Baptist Health Louisville

731	United States	Louisiana Heart Center
732	United States	Bryan Heart
734	United States	The Research Group of Lexington LLC
735	United States	Grace Research, LLC
736	United States	Grace Research, LLC_Shreveport
737	United States	University of Texas Southwestern Medical Center

Supplementary Table 1: Regression Analysis of Baseline BMI (Continuous) and Baseline Endpoint Assessments (Prior to Treatment)

	Predicted change per 1 kg/m^2 increase in Body Mass Index (BMI)			
	**Model 1 (Univariate)**		**Model 2***	
	**Slope (95% CI)**	***P***	**Slope (95% CI)**	***P***
KCCQ-CSS (points)	-0.84 [-1.08; -0.59]	<0.0001	-0.66 [-0.92; -0.40]	<0.0001
6MWD (m)	-4.52 [-5.76; -3.28]	<0.0001	-5.40 [-6.62; -4.19]	<0.0001
CRP (geometric mean ratio)	1.04 [1.03; 1.06]	<0.0001	1.03 [1.02; 1.05]	<0.0001
NTproBNP (geometric mean ratio)	0.99 [0.98; 1.01]	0.361	1.00 [0.99; 1.01]	0.743

Data are point estimates and 95% confidence intervals, computed using linear or multivariable regression analyses; *P* values are two-sided.
*Model 2 adjusted for baseline age, sex, history of atrial fibrillation, history of coronary artery disease, and NYHA class

6MWD, 6 minute walk distance; BMI, body mass index; CI, confidence interval; CRP, C-reactive protein; KCCQ-CSS, Kansas City Cardiomyopathy Questionaire Clinical Summary Score; ; NTproBNP, N-terminal pro-Brain natriuretic peptide; SBP, systolic blood pressure.
